# 262. Inhibiting macrophage Warburg effect blocks DNA hyper-methylation and restores immune function

**DOI:** 10.1093/ofid/ofad500.334

**Published:** 2023-11-27

**Authors:** Abhimanyu Abhimanyu, Malik Ladki, Daanish Sheikh, Tomoki Nishiguchi, Santiago Carrero Longlax, Andrew DiNardo

**Affiliations:** BCM, Houston, Texas; BCM, Houston, Texas; BCM, Houston, Texas; BCM, Houston, Texas; BCM, Houston, Texas; BCM, Houston, Texas

## Abstract

**Background:**

Severe and chronic infections, such as sepsis and Tuberculosis (TB), lead to epigenetic-mediated immune suppression. Cancer studies have demonstrated that perturbations in TCA cycle intracellular metabolites trigger global changes in the epigenetic landscape, resulting in immune suppression that persists after microbial eradication. Therefore inhibiting the TCA cycle may mitigate epigenetic-mediated immune suppression.

**Methods:**

We applied complementary approaches, including data mining public tuberculosis and sepsis datasets, an in vitro LPS-stimulated macrophage tolerance model, DNA methylation (Methyl EPIC), confocal microscopy, and reverse phase protein array (RPPA) to investigate mechanisms of post-infectious immune suppression. Results were validated in tuberculosis patients in a clinical trial using an mTOR inhibitor.

**Results:**

Patients with TB and sepsis both demonstrated increased expression of genes involved in glycolysis and the tricarboxylic acid (TCA) cycle, which correlated with an increase in DNA methylation. Human monocytes stimulated with LPS in vitro developed immune tolerance that correlated with DNA hypermethylation. LPS induced the TCA enzymes isocitrate dehydrogenase, citrate synthetase, as well as DNMT3b to translocate into the nucleus. Inhibition of TCA cycle overactivation blocked LPS-induced immune suppression and DNA hyper-methylation. Phase 2 TCA metabolites succinate and itaconate mimicked LPS-induced immune suppression and DNA hyper-methylation. In vitro studies were validated in vivo with TB patients receiving mTOR inhibition (everolimus) having less DNA hyper-methylation.

**Figure:**
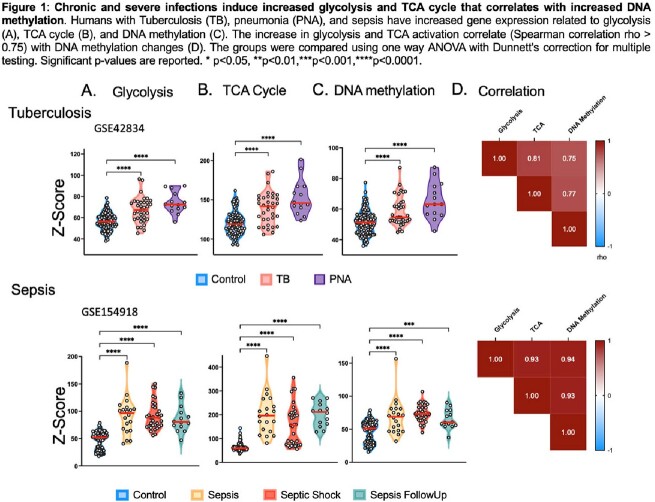

**Figure:**
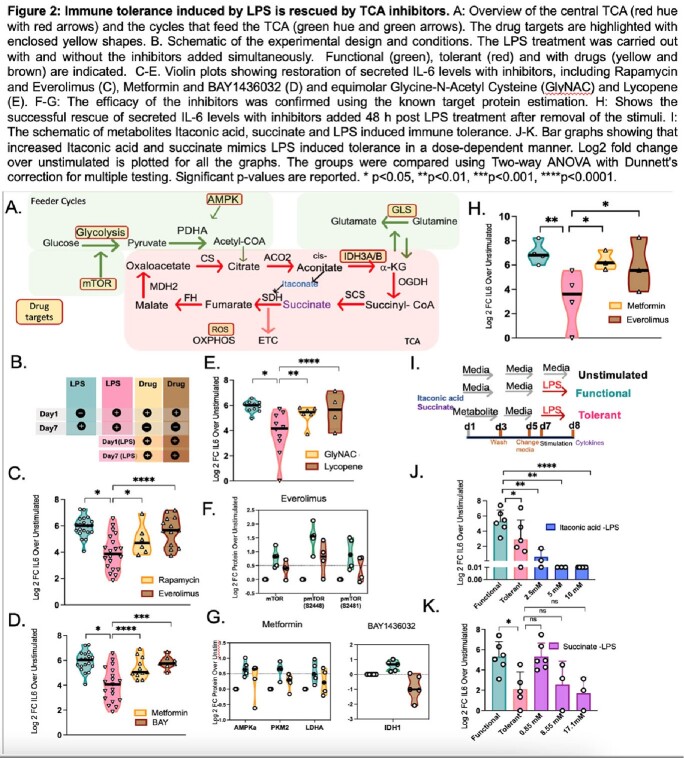

**Figure:**
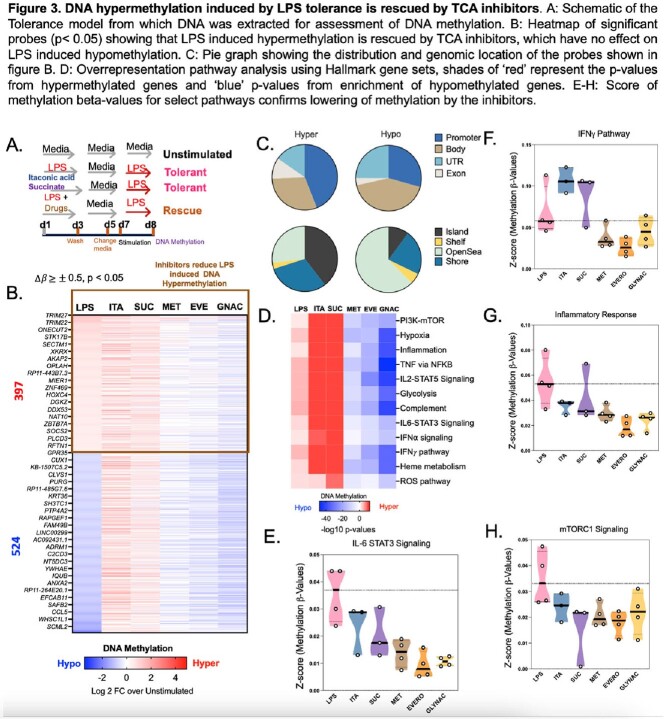

**Conclusion:**

TCA cycle activation regulates DNA methylation and immune tolerance. These in vitro and in vivo studies demonstrate that inhibiting the TCA cycle can mitigate epigenetic-mediated immune silencing, potentially identifying a means to prevent post-infectious immune suppression.

**Disclosures:**

**All Authors**: No reported disclosures

